# Exploring predictors of welfare dependency 1, 3, and 5 years after mental health-related absence in danish municipalities between 2010 and 2012 using flexible machine learning modelling

**DOI:** 10.1186/s12889-023-15106-y

**Published:** 2023-02-02

**Authors:** Søren Skotte Bjerregaard

**Affiliations:** The National Research Centre for the Working Environment, 105 Lersø Parkallé, DK-2100 Copenhagen, Denmark

**Keywords:** Welfare dependency, Return to work, XGboost, Machine learning, Shapley additive explanation, Common mental disorders

## Abstract

**Background:**

Using XGBoost (XGB), this study demonstrates how flexible machine learning modelling can complement traditional statistical modelling (multinomial logistic regression) as a sensitivity analysis and predictive modelling tool in occupational health research.

**Design:**

The study predicts welfare dependency for a cohort at 1, 3, and 5 years of follow-up using XGB and multinomial logistic regression (MLR). The models’ predictive ability is evaluated using tenfold cross-validation (internal validation) and geographical validation (semi-external validation). In addition, we calculate and graphically assess Shapley additive explanation (SHAP) values from the XGB model to examine deviation from linearity assumptions, including interactions. The study population consists of all 20–54 years old on long-term sickness absence leave due to self-reported common mental disorders (CMD) between April 26, 2010, and September 2012 in 21 (of 98) Danish municipalities that participated in the Danish Return to Work program. The total sample of 19.664 observations is split geospatially into a development set (*n* = 9.756) and a test set (*n* = 9.908).

**Results:**

There were no practical differences in the XGB and MLR models’ predictive ability. Industry, job skills, citizenship, unemployment insurance, gender, and period had limited importance in predicting welfare dependency in both models. On the other hand, welfare dependency history and reason for sickness absence were strong predictors. Graphical SHAP-analysis of the XGB model did not indicate substantial deviations from linearity assumptions implied by the multinomial regression model.

**Conclusion:**

Flexible machine learning models like XGB can supplement traditional statistical methods like multinomial logistic regression in occupational health research by providing a benchmark for predictive performance and traditional statistical models' ability to capture important associations for a given set of predictors as well as potential violations of linearity.

**Trial registration:**

ISRCTN43004323.

## Background

The significant growth in data across a broad range of fields, including biology, medicine, finance, marketing, and epidemiology, has paved the way for modern data mining techniques to extract important patterns and trends [1, 2]. The potential of machine learning for data-driven examination of predictive patterns is gaining more traction among colleagues in occupational health research. Therefore, a demonstration of these novel tools and clarification on how these tools relate to more traditional approaches in occupational health research and epidemiology is warranted. The novelty of machine learning lies in its ability to model associations in data in a more automated fashion with fewer restrictions than a researcher explicitly or implicitly makes with a traditional regression approach. Machine learning can thus help the researcher to consider associations that the researcher would not otherwise have. Further, machine learning offers an alternative tool for assessing predictor importance and sensitivity analysis.

### Data-driven predictive modelling with machine learning

Machine learning is commonly applied in prediction problems or as an exploratory tool where inference about specific parameters is less critical. Flexible machine learning models can have high predictive performance across various applications [3]. However, they are often considered “black box” models because the relationship between the predictors and the outcome can be challenging to assess. Thus, even if flexible machine learning models can achieve better predictive ability, the researcher will often need to build simpler models that are easier to interpret. That could, for example, be a traditional regression model, which provides readily interpretable parameters or, in some cases, after relatively simple transformations.

To contrast the two approaches, a flexible machine learning model will, in an automated fashion, attempt to find optimal predictive associations between the outcome and predictors, including interaction. Further, flexible machine learning models might find predictive associations the researchers have not thought of or find too challenging to model with a traditional regression approach [2]. On the other hand, a traditional regression approach needs to specify new models with interaction terms and rerun the regressions. Further, the traditional regression models will generally rely on stronger assumptions than flexible machine learning models. However, even for researchers that take the traditional regression approach, flexible machine learning models provide a helpful benchmark for the predictive ability of simpler models and allow the researcher to balance predictive ability against interpretability [4]. For example, suppose a flexible machine learning model has significantly better predictive performance than a traditional regression model. This difference could indicate that the latter model does not adequately capture the underlying data mechanism [5]. Further, this could mean violations of important assumptions in linear models, such as linearity and additivity, which can impair predictive performance.

There is, however, an alternative to the paradigm of complex models first followed by simpler and more interpretable models. The researcher can stick with a highly predictive “black-box” model and use explanatory algorithms to extract how the predictors in a “black-box” model contribute to the predicted outcome. We illustrate the below by deriving SHAP-values from a “black-box” model.

### A practical demonstration of flexible machine learning against traditional regression

The remaining part of the present paper will demonstrate how flexible machine learning can be applied for.

1. Predictive modelling, and
2. sensitivity analysis and model adequacy assessment (does more flexible modelling improve model fit?)

For this demonstration, we use a case study to identify important predictors of welfare dependency at follow-up after 1, 3, and 5 years for individuals on long-term sickness absence leave due to common mental disorders (not necessarily work-related psychological injuries). Welfare dependency makes an attractive outcome because data lets us observe welfare transfers weekly. This property allows us to evaluate the outcome at follow-up with high precision and minimum bias compared to data relying on monthly or yearly status. Moreover, welfare dependency is a proxy for return to work (RTW), assuming that most persons no longer on welfare have returned to work. A few papers use machine learning to predict RTW (e.g. [6, 7]), and RTW from CMD has already received considerable attention in academic research (e.g. [8–13]). Therefore, we relate our results to studies that use RTW as the outcome, although we recognize that it is not always the case that persons no longer on welfare have returned to work.

National statistics show that the share of persons with a long-term sickness absence spell to all employed was around 5–6 pct. (130.000–160.000 persons) in the year 2010–2012. Our data shows that about a third self-report mental health as the reason for long-term sickness absence. These numbers suggest that around 2 pct. of all employed (44.000–54.000 persons) each year in 2010–2012 experienced a long-term sickness absence spell due to CMD. Additionally, mental health disorders are increasing in Denmark across all working-age groups [14]. Thus, common mental disorders (CMD) affect many workers and are likely to be an increasing problem if current trends persist. This scenario makes it increasingly interesting to determine important predictors of welfare dependency from CMD. In addition, predicting individualized welfare dependency risk can help job centres prioritize efforts on high-risk individuals rather than those already likely to return to work. Further, prediction models can help distribute resources across job centres within municipalities. For example, suppose a disproportional distribution of individuals at high risk for prolonged welfare dependency across job centres. In that case, municipalities can direct resources to job centres with higher proportions of high-risk individuals.

Flyvholm and Hannerz [8] describe a protocol that examines important predictors in welfare dependency using multinomial logistic regression (MLR). The present paper considers the same data set and demonstrates how the popular machine learning model XGBoost (XGB) can complement MLR to assess model adequacy, provide sensitivity analysis, and improve the predictive relationship between predictors and the outcome.

At least two previous papers apply ML with Korean survey data to predict RTW following sickness absence [6, 7], which closely relates to the concept of welfare dependency. As an additional contribution to existing papers, the present paper provides a benchmark for predictive performance using administrative data. The reliance on administrative data limits the set of predictors. However, administrative data is often relatively cheap to collect, and some administrative data sets can reduce measurement errors and bias related to self-reports. Further, the present paper contributes to the existing literature by supplementing internal validation with geographical validation for external validation. The existing papers only use internal validation techniques (e.g. tenfold cross-validation), but external validation is considered to provide a better measure of the models’ generalizability to new settings [3]. For example, external validation likely gives a better estimate of how well we can expect the models to predict welfare dependency in the municipalities that were not included in this study. Lastly, this paper contributes to the existing literature by demonstrating how the explanatory algorithm SHAP can help to examine the predictive patterns from the “black-box” machine learning models.

## Methods

The present paper takes the following three-step approach to demonstrate how XGB can complement MLR:

- First, benchmark the predictive ability of a multinomial logistic regression model against an XGB model in predicting welfare dependency for individuals on sickness absence due to CMD at 1, 3, and 5 years follow-up. This step is critical to ascertain that XGB can reliably capture the underlying data mechanism.
- Second, examine the importance of the predictors across the two modelling approaches to see whether the models agree on what predictors are more important.
- Third, explore whether the XGB models suggest patterns that deviate from the MLR. This can provide guidance for new model specifications for MLR that can improve its predictive ability.

### Study population

The study population consists of all 20–54 years old on long-term sickness absence leave due to self-reported CMD between April 26, 2010, and September 2012 in 21 (of 98) Danish municipalities that participated in the Danish Return to Work program [15]. The original study collected data from jobs and benefits offices in each participating municipality. Additional variables have been added subsequently from national registries. We ascertain both deaths and emigration using the Central Person Registry [16].

**Fig. 1:**
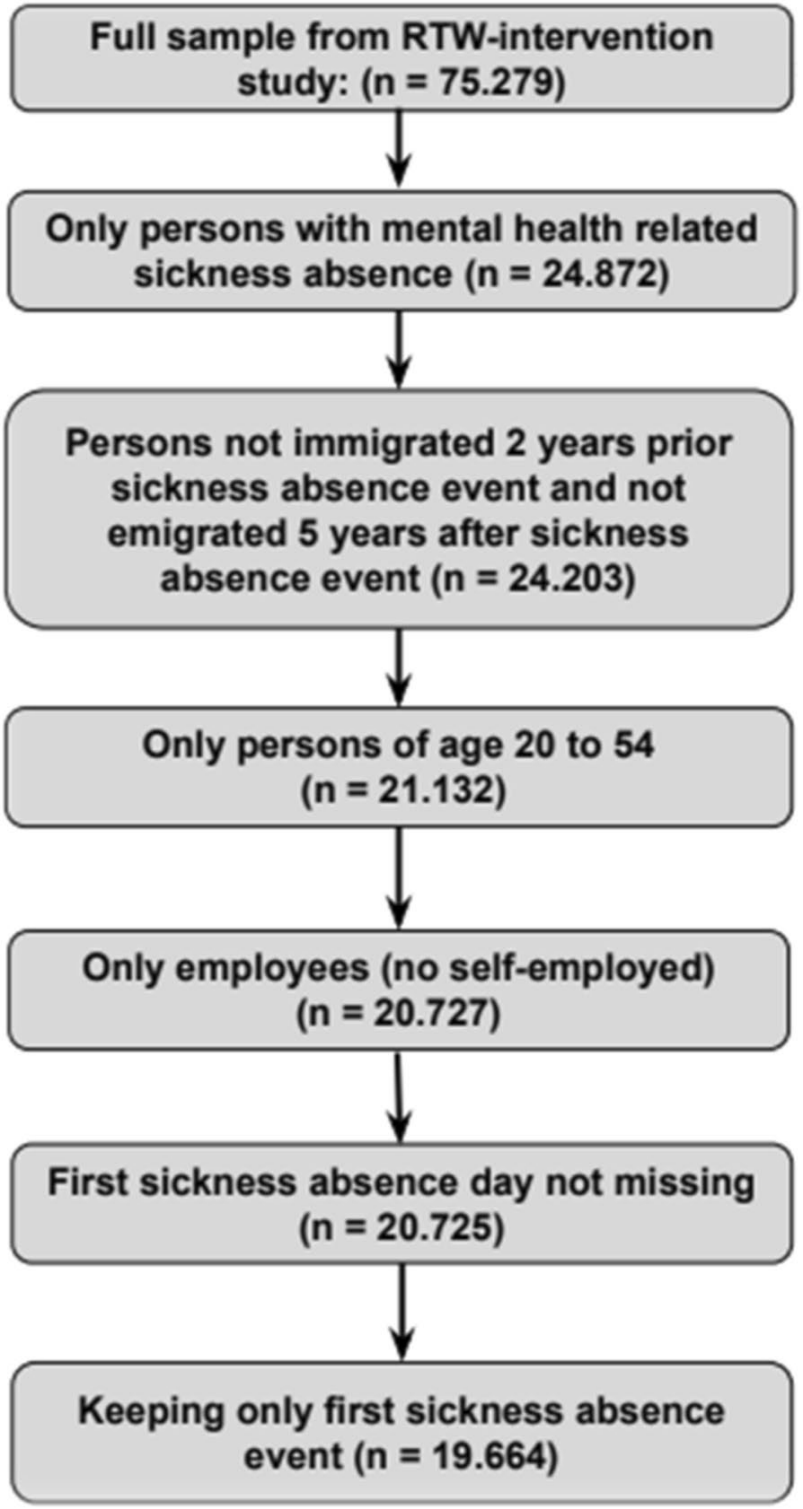
Participant flow diagram

Only records with self-reported CMD sickness absence (depression, anxiety, stress/burnout, or mental ill-health without further specification) are kept from the original sample from the RTW program. We restrict the sample to persons that immigrated less than two years prior to the sickness absence event. This restriction ensures that information on predictors for the number of weeks with social benefits or health-related benefits two years prior to the sickness absence spell is consistent for all observations. Further, to be able to examine welfare dependency at follow-up, we remove persons that emigrated less than 5 years after the sickness absence event. Also, the sample is restricted to employees of ages 20–54 to avoid employees reaching 60 during follow-up, where some workers are eligible for early-retirement schemes. Further, we want to examine the predictive importance of job skill level and therefore remove self-employed, which is not assigned a job skill level in administrative data. Removing self-employed reduces the sample size by 4 pct. Additionally, we remove records with a missing first sickness absence day. Finally, for workers with several sickness-absence spells in the data, we only use the workers once with the predictor values that associate with the first sickness-absence spell. Figure 1 shows the sample size implications in a participant flow diagram.

Table 1 presents the variables Flyvholm and Hannerz [8] described in their protocol and collected after reviewing the literature. The table provides brief descriptions of predictors, the expected predictive association, the predictive association's source, and the variable's data source in the present study. Below we describe a few of the variables that require further elaboration.

**Table 1:** Variables, Expected predictive association, and sources

Variable	Expected predictive association	Source	Data source
**Outcomes**			
Health benefit recipient Recipient of other benefits Not benefit recipient			The Danish Register for Evaluation of Marginalisation
**Predictors**			
Job group skill level (last recorded during a two-year period preceding baseline)	Faster RTW for higher job skill level	[12, 13, 17]	Income Statistics Registry
Industrial sector (last recorded during a two-year period preceding baseline)	Slower RTW for the educational and training industry	[11]	Employment Classification Module
Reason for sickness absence (self-reported)	Slower RTW for increasing severity	[9]	Local jobs and municipality offices
Gender	Inconclusive	[9]	Central Person Registry
Age (at baseline)	Slower RTW for higher age	[9]	Central Person Registry
Family type (in the calendar year preceding baseline)	Slower RTW for having a partner No reports for children	[9]	Central Person Registry
Employed at baseline (vs unemployed)	Faster RTW for employed	[18]	Local jobs and municipality offices
Unemployment insurance (in the calendar year preceding baseline)	Inconclusive due to selection effect of UI	[19]	The Danish Register for Evaluation of Marginalisation
Danish citizenship	Faster RTW		Central Person Registry
Calendar year (at the start of the concerned sickness absence episode)	None		Local jobs and municipality offices
Time passed between the first day of sickness absence and the baseline visit at the jobs and benefits office	Slower for longer TTV	[20]	Local jobs and municipality offices
Participation in Danish RTW-program	None		Local jobs and municipality offices
Number of weeks with healt- related social transfer payments during a two-year period prior to the baseline sickness absence episode	Slower RTW for more history as health benefit recipient	[9]	The Danish Register for Evaluation of Marginalisation
Number of weeks with other social transfer payments (except for state educational grants and maternity/paternity leave benefits) during a two-year period prior to the baseline sickness absence episode	Slower RTW for more history as other social benefit recipient	[18]	The Danish Register for Evaluation of Marginalisation
**Data-split variable**			
Geographical region			Local jobs and municipality offices

Data access is limited to researchers that have been granted access to Danish administrative data through Statistics Denmark’s research server

The outcome is one of the following three categories:

1. Health benefits recipient
2. Recipient of other benefits
3. Not benefit recipient

A health benefit recipient is defined as a deceased or a recipient of health-related social transfers. A recipient of other benefits is defined as one that receives other than health-related benefits. Aside from benefits designed mainly for preventing income loss, both groups 1 and 2 also include subsidized job training programs. Persons in these job training programs still depend on welfare benefits and are therefore assigned to these groups depending on the specific transfer they receive. No benefits recipients are defined as individuals who did not receive social transfer payments, with a few exceptions. We also assign maternity/paternity leave, state education grants, and holiday allowance to the “Not benefit recipient”-category. The mapping between DREAM-codes and the outcome categories is available in Table 2.

**Table 2:** Mapping between outcome groups and DREAM-codes

Outcome category label	DREAM-code
Not benefit recipient	881
	651, 652, 661
	121 Empty field
Health benefit recipient	750, 753, 754, 755, 756, 757, 758
	760, 763, 764, 765, 766, 767, 768
	771, 775, 781,783, 785
	810, 813, 814, 815, 816, 817, 818
	890, 893
Other benefit recipient	All other codes

### Welfare dependency history

The DREAM registry provides weekly information on the benefit transfer a person receives. Benefit transfer history is likely important information in predicting future benefit transfers. We reason that higher past reliance on health benefits is a strong predictor of reliance on health benefits at follow-up. In turn, past reliance on other benefits is likely a strong predictor for other social benefits at follow-up. Therefore, we construct two predictors from welfare dependency history:

1. The number of weeks receiving health-related benefits during the period two years prior to the sickness absence episode.
2. The number of weeks receiving other social benefits during the period two years prior to the sickness absence episode.

These variables match the DREAM-codes of the outcomes “Health benefit recipient” and “Other benefit recipient”, respectively.

### Predictors from municipality jobs and benefit offices

The Danish public sickness benefits scheme covers employed, unemployed, self-employed, and assisting spouses with long-term sickness benefits absence (> 21 days in 2010–2011, > 30 days in 2012). Municipal jobs and benefits offices administer the system and are committed to following up and evaluating sick-listed persons' prognosis of return to the labour force continuously [21]. In relation to the study, the Danish Return to Work program, 21 out of 98 participating municipalities collected data between April 26 2010 and September 2012. This data contains the date of the first visit to a jobs and benefits office, the date of the start of the sickness absence episode, the self-reported reason for the sickness absence, RTW-intervention status, geographical region, employment status, and personal identification number. The latter enables linkage to data in national registers.

The predictor, *“participation in Danish RTW intervention”,* is divided into three groups: intervention, control, and not eligible. The intervention and control group consists of persons who are not expected to RTW within 3 months but are considered to gradually RTW or participate in RTW activities. The group “not eligible” contain both persons considered able to RTW within 3 months and persons that are not able to RTW within 3 months nor able to gradually RTW or participate in RTW activities due to serious illness, hospitalization or the like [15].

*Geographical region* is not included as a control variable, as is the case in the protocol by [8]. We use the geographical region to split data into a development set and a test set. This prohibits us from using the geographical region as a predictor.

Table 3 provides descriptive statistics for both the development and test set.

**Table 3:** Descriptive statistics

Characteristic	Development, *N* = 9,756	Test, *N* = 9,908
Status 1 year after sickness absence leave		
Not benefit recipient	5,517 (57%)	5,253 (53%)
Other benefit recipient	2,333 (24%)	2,095 (21%)
Health benefit recipient	1,906 (20%)	2,560 (26%)
Status 3 years after sickness absence leave		
Not benefit recipient	6,319 (65%)	5,827 (59%)
Other benefit recipient	2,049 (21%)	2,019 (20%)
Health benefit recipient	1,388 (14%)	2,062 (21%)
Status 5 years after sickness absence leave		
Not benefit recipient	6,487 (66%)	5,965 (60%)
Other benefit recipient	1,588 (16%)	1,491 (15%)
Health benefit recipient	1,681 (17%)	2,452 (25%)
Age	38 (31, 45)	38 (31, 45)
Age groups		
20–29	1,830 (19%)	2,020 (20%)
30–39	3,531 (36%)	3,493 (35%)
40–49	3,261 (33%)	3,192 (32%)
50–54	1,134 (12%)	1,203 (12%)
Time passed between the first day of sickness absence and the baseline visit at the jobs and benefits office	55 (42, 64)	45 (35, 56)
Time passed between the first day of sickness absence and the baseline visit at the jobs and benefits office (grouped)		
< 30	1,026 (11%)	1,765 (18%)
31–60	5,616 (58%)	6,616 (67%)
> 60	3,114 (32%)	1,527 (15%)
Period		
Before 2012	7,803 (80%)	8,076 (82%)
2012	1,953 (20%)	1,832 (18%)
Family type in the calendar year preceding the baseline		
Couple with resident children	3,790 (39%)	4,843 (49%)
Couple without resident children	1,505 (15%)	1,670 (17%)
Single with resident children	1,471 (15%)	1,182 (12%)
Single without resident children	2,986 (31%)	2,213 (22%)
Missing	4 (< 0.1%)	0 (0%)
Weeks receiving health-related social benefits during a two-year period prior to the baseline of sickness absence episode	0 (0, 7)	0 (0, 9)
Weeks receiving health-related social benefits during a two-year period prior to the baseline of sickness absence episode (grouped)		
A: 0 weeks	5,738 (59%)	5,443 (55%)
B: 1–26	3,064 (31%)	3,226 (33%)
C: > 26	954 (9.8%)	1,239 (13%)
Weeks receiving non-health related social benefits during a two-year period prior to the baseline of sickness absence episode	0 (0, 16)	0 (0, 17)
Weeks receiving non-health related social benefits during a two-year period prior to the baseline of sickness absence episode (grouped)		
A: 0 weeks	5,846 (60%)	5,757 (58%)
B: 1–26	1,959 (20%)	2,226 (22%)
C: > 26	1,951 (20%)	1,925 (19%)
Sickness absence reason		
Anxiety	379 (3.9%)	289 (2.9%)
Depression	3,693 (38%)	4,449 (45%)
Mental ill health without further specification	1,028 (11%)	1,169 (12%)
Stress/burnout	4,656 (48%)	4,001 (40%)
Skill level		
Highest	1,252 (13%)	1,216 (12%)
Medium	2,207 (23%)	2,348 (24%)
Basic	4,386 (45%)	4,457 (45%)
Few or no	1,066 (11%)	1,018 (10%)
Unstated	845 (8.7%)	869 (8.8%)
Industry		
Accommodation and food service activities	299 (3.1%)	274 (2.8%)
Agriculture	100 (1.0%)	106 (1.1%)
Construction	591 (6.1%)	621 (6.3%)
Courts and prisons, Police, Fire Departments	111 (1.1%)	113 (1.1%)
Education	731 (7.5%)	842 (8.5%)
Human health and social work activities	2,596 (27%)	2,761 (28%)
Manufacturing, mining and quarrying	893 (9.2%)	888 (9.0%)
Other branches	2,288 (23%)	2,110 (21%)
Public administration	336 (3.4%)	306 (3.1%)
Transporting and storage	548 (5.6%)	553 (5.6%)
Unstated	47 (0.5%)	54 (0.5%)
Wholesale and retail trade, repair of motor vehicles and motorcycles	1,216 (12%)	1,280 (13%)
Unemployment insurance (in the calendar year preceding the baseline)		
Insured	8,413 (86%)	8,716 (88%)
Not insured	1,343 (14%)	1,192 (12%)
Citizenship		
Danish	9,188 (94%)	9,544 (96%)
Not Danish	564 (5.8%)	364 (3.7%)
Missing	4 (< 0.1%)	0 (0%)
Participation in the Danish RTW program		
Control	1,771 (18%)	2,075 (21%)
Intervention	2,568 (26%)	2,241 (23%)
Not eligible	5,417 (56%)	5,592 (56%)
Employment status at baseline		
Employed	7,479 (77%)	7,670 (77%)
Unemployed	2,277 (23%)	2,238 (23%)
Gender		
Men	6,886 (71%)	6,836 (69%)
Women	2,870 (29%)	3,072 (31%)

*n* (%); Median (IQR)

### Statistical methods

We trained two learners to predict welfare dependency, namely a multinomial logistic regression and an XGBoost model. XGboost is one of many flexible machine learning models but has several beneficial “off-the-shelf” properties that make it apt for the present application. Most importantly, XGboost is easy to model and have been shown to have high predictive performance across several different application. In addition, we computed SHAP-values for the XGB models to assess the impact of each variable in the models on the predicted outcome to help us interpret the models.

#### Multinomial logistic regression (MLR)

Multinomial logistic regression is a generalization of binary logistic regression that adapts to situations with multiple outcome categories but no natural ordering [22]. The multinomial regression is performed in R using the nnet-package [23].

#### XGBoost (XGB)

XGB belongs to the class of boosting algorithms that starts with a weak model and then sequentially boosts performance by adding new models that try to fix the mistakes made by previous models. This forms an ensemble of models that combines results into a single prediction [24]. XGB based on decision trees inherits the advantages of this class of learners. Thus, XGB can incorporate a mixture of continuous and categorical variables. Further, XGB is invariant under strictly monotone transformation of individual predictors, insensitive to outliers, and internally performs feature selection. By boosting low-accuracy decision trees, boosting algorithms can increase performance dramatically without sacrificing many of the benefits of decision trees [1]. These “off-the-shelf” properties make XGB useful for various problems. 

Table 4 displays the hyperparameters applied in a grid search, indicating the scope of different XGB-models cross-validated before deciding on a final model. XGB is trained in R using the XGboost-package [25].

**Table 4:** Hyperparametergrid for XGBoost

nrounds	100 to 1.000 by 100
eta	0.025, 0.05, 0.1, 0.3
max_depth	2, 4, 6
gamma	0
colsample_bytree	1
min_child_weight	1
subsample	1

XGBoost grid from XGBoost package in R

#### SHapley Additive eXplanation (SHAP)

The SHAP value estimates the impact of each variable on the predicted outcome based on game theory, where each predictor is considered a player. SHAP fairly attributes predictive performance to each variable, which explains each predictor's contributions for a single observation. The observation-specific SHAP-values can be averaged and evaluated using graphical SHAP summary plots to assess overall predictor importance. In addition, SHAP dependence plot can evaluate non-linear effects of predictors. We will use this approach to interpret how XGB predicts welfare dependency. Other studies have used a similar approach to interpret the prediction mechanism of XGB in breast cancer survival [26], melanoma risk prediction [27], and freight truck related crashes [28]. SHAP values are computed using the XGBoost-package.

#### Bagged trees

We use the bagged trees algorithm for imputation. With this algorithm, we create a decision tree for each of 5 bootstrap samples using all predictors. The bagged trees then impute the majority predicted class for categorical values and the mean of continuous values. This imputation algorithm is more powerful but also more computationally expensive than median imputation [4] but relatively easy to implement with the caret-package that we use.

### Missing data

Job group skill level and industrial sector were the only variables with a relatively large fraction of missing values. These variables are not registered for unemployed unless a person has been employed at some point during the year. For employed, this information can be missing because small companies (< 10 employees) are not required to register the information on which these variables are based. To deal with this, we impute job group skill level or industrial sector using last observation carried forward going a maximum of two years back in time. The remaining missing values are categorized as unstated (8.7 pct. for job skill level and 0.5 pct. for industrial sector). However, there is still a risk that this strategy can lead to suboptimal prediction if low job skills are overrepresented in the “unstated” group. Therefore, we also test whether model-based single imputation with bagged trees can improve the predictive ability to the dummy-based strategy. Case-wise deletion was applied to four observations with missing family type and citizenship.

### Assessment of predictive ability

We evaluate apparent performance (training and validation on development data) and the mean of tenfold cross-validation for internal validation. To this end, we use the caret-package in R [29]. We use a cross-validation procedure to reduce the risk of overfitting data and thereby improve the models’ ability to generalize to new “unseen” data. In practice, we train the model on 9 folds of the training data and evaluate model performance on the remaining fold of training data. We repeat this procedure 10 times, so each fold is used 9 times to train the model and once for model evaluation. We evaluate external performance by splitting the data by geographical location. The model is developed on data from two regions (Capital and Zealand), and external validation is performed on the test set comprising three other regions (Southern Denmark, Central Jutland, and Northern Jutland). This approach provides a better estimate of external validity to procedures that use a random split [3].

The Brier score assesses the models’ overall performance. Higher Brier scores associate with worse performance. The Brier score range between 0 and a maximum score that depends on the incidence in the outcome groups. Therefore, we report the scaled Brier score, which indicates the improvement over the maximum Brier score. The maximum Brier score is the Brier score from a model that predicts the average values for each outcome category in the training data. For example, at 3 years follow-up, the maximum Brier score is based on predicting 65 pct. for “Not benefit recipient”, 21 pct. for “Other benefit recipient”, and 14 pct. for “Health benefit recipient” for all observations. The scaled Brier score is on a scale from 0–1, where 0 represents no improvement, and 1 represents perfect prediction. Thus, higher values are better on the scaled Brier score. The Area Under the Curve Receiver Operating Characteristics (AUC-ROC) assesses discrimination, the models' ability to assign a higher probability to events vs non-events. AUC-ROC ranges between 0.5 (no ability to discriminate) and 1 (perfect discrimination). As a rule of thumb, the discrimination for AUC-ROC between 0.5–0.7 is considered poor, 0.7–0.8 is acceptable, 0.8–0.9 is excellent, and > 0.9 is outstanding [30]. However, these cut-points are a bit arbitrary and more conservative accounts only consider models with values above 0.8 to be useful [31]. Finally, we examine calibration, which helps to evaluate how closely the risk prediction of a model agrees with the observed risk [31]. We assess calibration visually where the calibration curve is smoothed using cubic splines.

### Predictor importance

We evaluate predictor importance from MLR by calculating ${\chi }^{2}$ minus degrees of freedom from a likelihood ratio test that compares the full model with the full model minus the predictor of interest. For XGB, we evaluate predictor importance by the mean SHAP-value for each outcome.

### Data mining of predictive associations from XGB using SHAP

We examine deviations from linearity by inspecting partial and two-way plots of SHAP values against predictors. Data mining may imply that some of these patterns are not generalizable beyond the development data.

## Results

Table 5 shows that validation results are largely similar for XGB and MLR across performance measures and types of validation for any given follow-up year. The AUC scores are very similar and around 0.7 for both models across the different follow-up periods. By some standards, 0.7 is acceptable, but neither models are great at discriminating outcomes. The scaled-Brier scores indicate that the models improve the predictive performance over the maximum Brier score in the range of 0.08–0.15. This shows that modelling can improve predictive performance but also that it is difficult to improve substantially.

**Table 5:** Validation results

Follow-up	1 year				3 years				5 years			
Model	MLR	PMLR_im_	XGB	XGB_im_	MLR	PMLR_im_	XGB	XGB_im_	MLR	PMLR_im_	XGB	XGB_im_
AUC-ROC												
Apparent	0.71	0.71	0.73	0.72	0.70	0.70	0.72	0.72	0.71	0.70	0.72	0.72
tenfold-cv	0.71	0.71	0.71	0.71	0.69	0.70	0.70	0.69	0.70	0.70	0.70	0.70
Test set	0.71	0.70	0.71	0.71	0.70	0.70	0.71	0.71	0.70	0.70	0.71	0.70
Brier-scaled												
Apparent	0.13	0.13	0.15	0.15	0.10	0.10	0.12	0.12	0.10	0.08	0.10	0.12
tenfold-cv	0.11	0.11	0.13	0.13	0.09	0.09	0.10	0.10	0.10	0.10	0.10	0.10
Test set	0.12	0.12	0.14	0.14	0.12	0.12	0.13	0.12	0.12	0.12	0.10	0.12

*MLR* Multinomial logistic regression, *PMLR*_im_ Penalized multinomial logistic regression (ridge penalty) with single imputation, *XGB* XGBoost, *XGB*_im_ XGBoost with single imputation

We also tried to improve the performance of the models by applying single imputation with bagged trees. In addition, we applied a ridge penalty to the coefficient (from 0–1 by 0.1) in the MLR models. Although models with some penalty did better than none, the improvements were too small to detect with two decimal points. This is not too surprising since penalized regression is more important when models are based on small datasets [3]. Imputation also made no discernable difference. We only imputed values for job skill level and industry, but, as we present briefly, these predictors have very limited importance. Therefore, we focus on the results for the XGB and MLR without penalty and imputation.

**Fig. 2:**
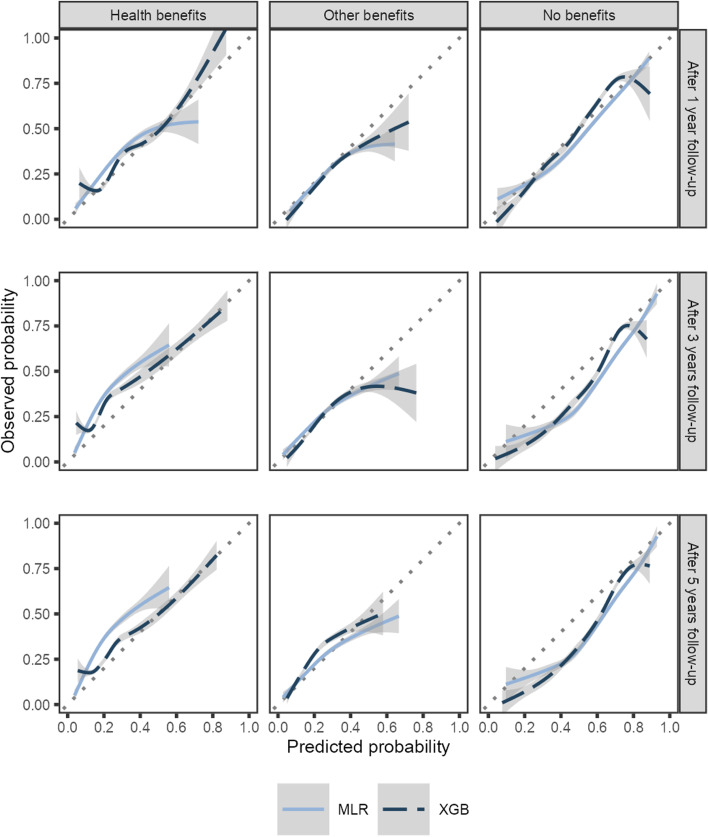
Calibration (agreement between observed and predicted probability smoothed using cubic splines)

Figure 2 shows the calibration curves smoothed using cubic splines for XGB and MLR for each model at each follow-up period on the test set. When the calibration curve is below the 45-degree line, the model assigns a too high probability of an event (overpredicts). In contrast, the model assigns a too low probability of an event (underpredicts) if the calibration curve is above the dotted line [31]. For recipients of other benefits, both MLR and XGB are well-calibrated where the density of data is high (confidence intervals narrow) after 1 and 3 years of follow-up. However, after 5 years of follow-up, the MLR overpredicts increasingly for higher predicted probabilities. MLR under-predicts the risk of being on a health benefits recipient and overpredicts the probability of not receiving benefits and increasingly so at longer follow-up. XGB underpredicts the risk of being a health benefit recipient at 3 and 5 years follow-up for predictions in the range of 0 to 40–50 pct. but to a lesser degree than MLR. However, at 3 and 5 years follow-up, XGB overpredicts not being a benefit recipient to the same degree as MLR. From Table 3, we note that the test set generally has a higher share of health benefit recipients and a smaller share of non-benefit recipients at follow-up than the development set, which might explain these results. This emphasizes the need for (semi-)external validation to get a realistic assessment of the model’s validity.

### Predictor importance

**Fig. 3:**
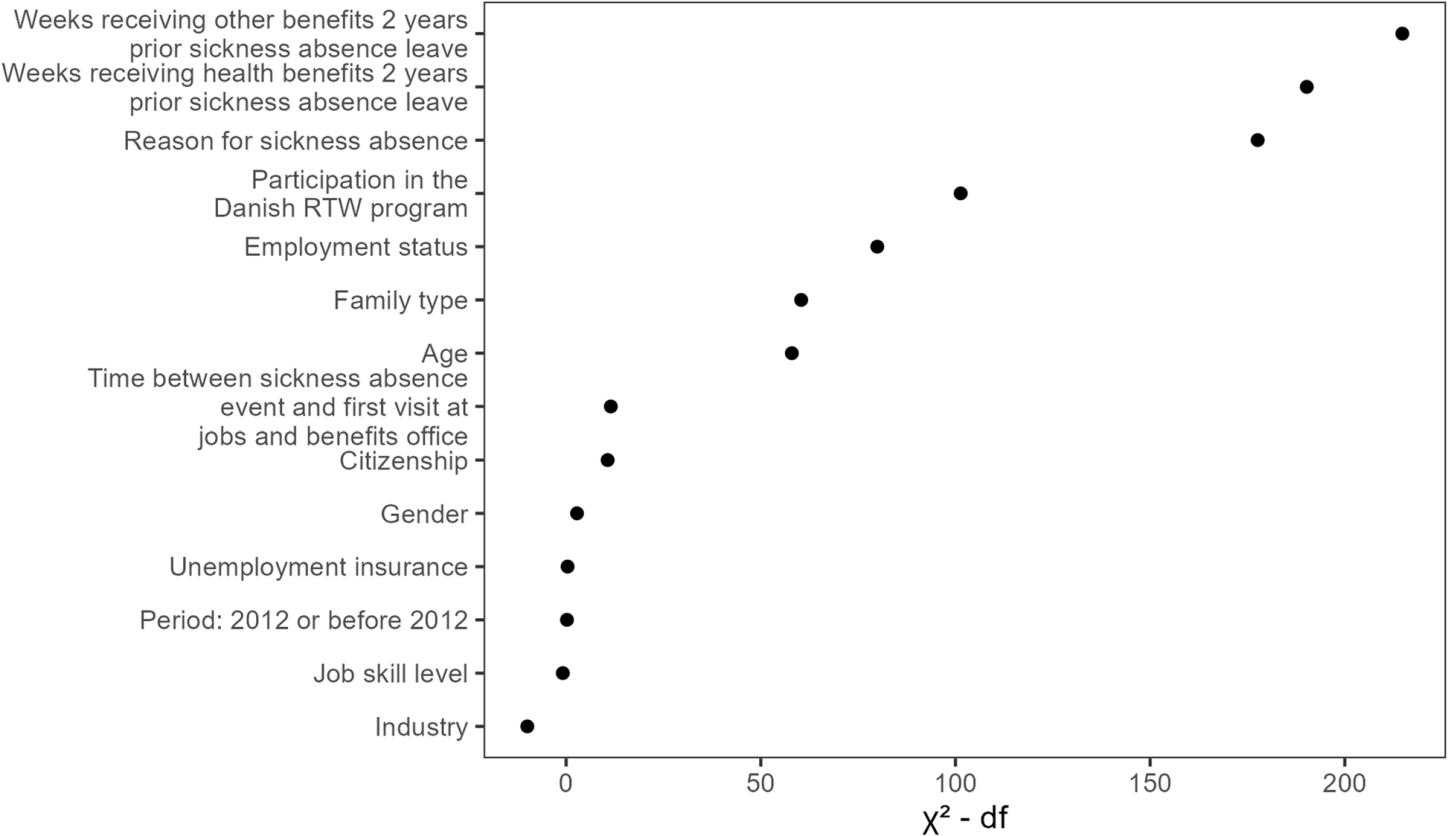
Predictor importance for MLR

Figure 3 shows the results at 1 year follow-up where larger values of ${\chi }^{2}$ minus degrees of freedom associate with larger importance. The two predictors with welfare dependency history are the most important, followed by reason for sickness absence. Industry, job skill level, period, unemployment history and gender are the least important predictors.

**Fig. 4:**
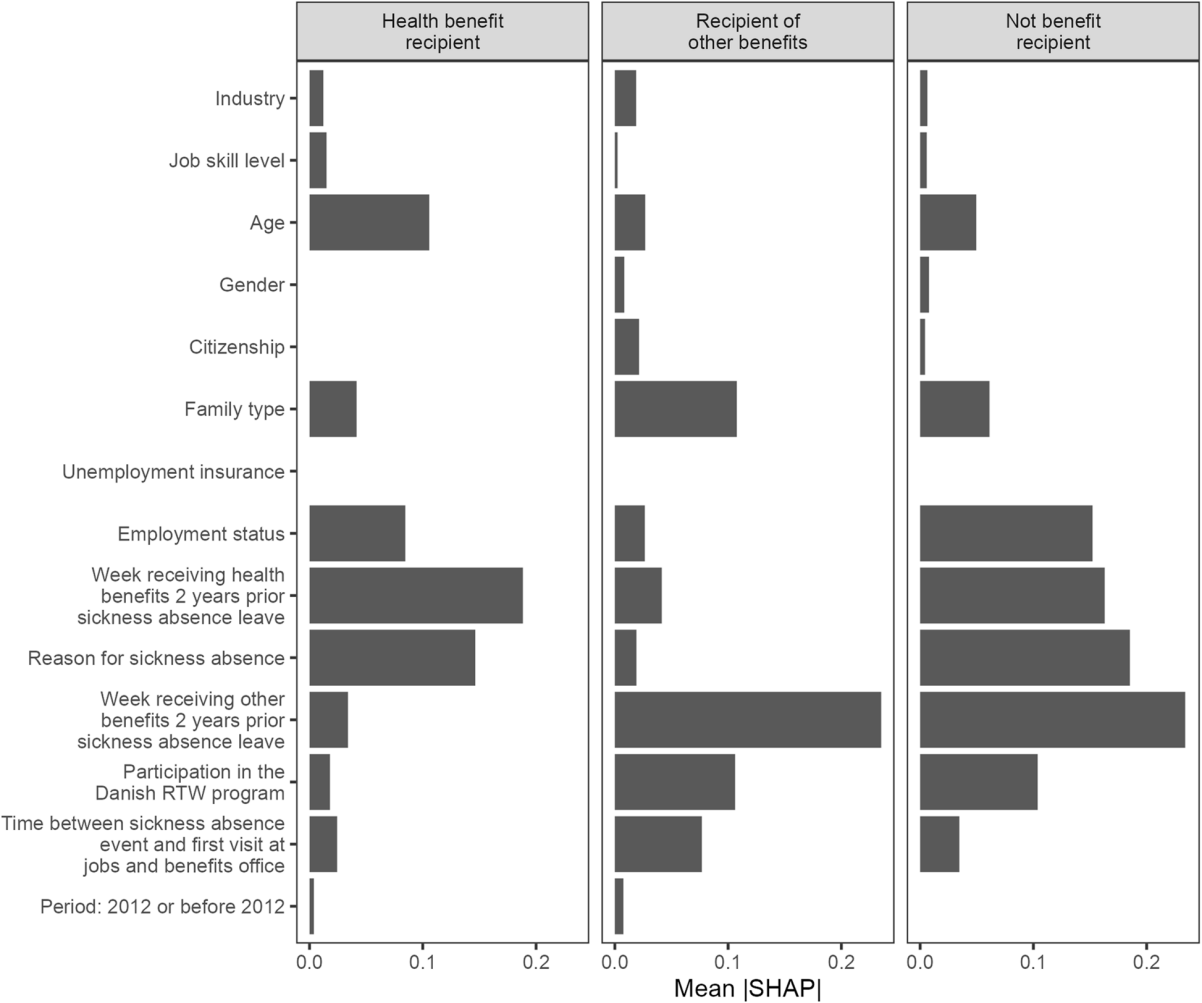
Predictor importance for XGB at 1 year follow up

For XGB, we visualize the mean absolute SHAP value. The larger this value, the larger the impact of the predictor in the prediction model. Figure 4 illustrates that predictors are not equally important in predicting the type of outcome. However, the overall pattern is that past welfare dependency and reason for sickness absence remain among the most important predictors. Job skill level and industry rank higher in the XGB-model compared to MLR but remain less important predictors alongside gender, period, unemployment insurance, and citizenship. Similar figures for 3 and 5 years follow-up corroborate this finding (not shown).

### Data mining results

**Fig. 5:**
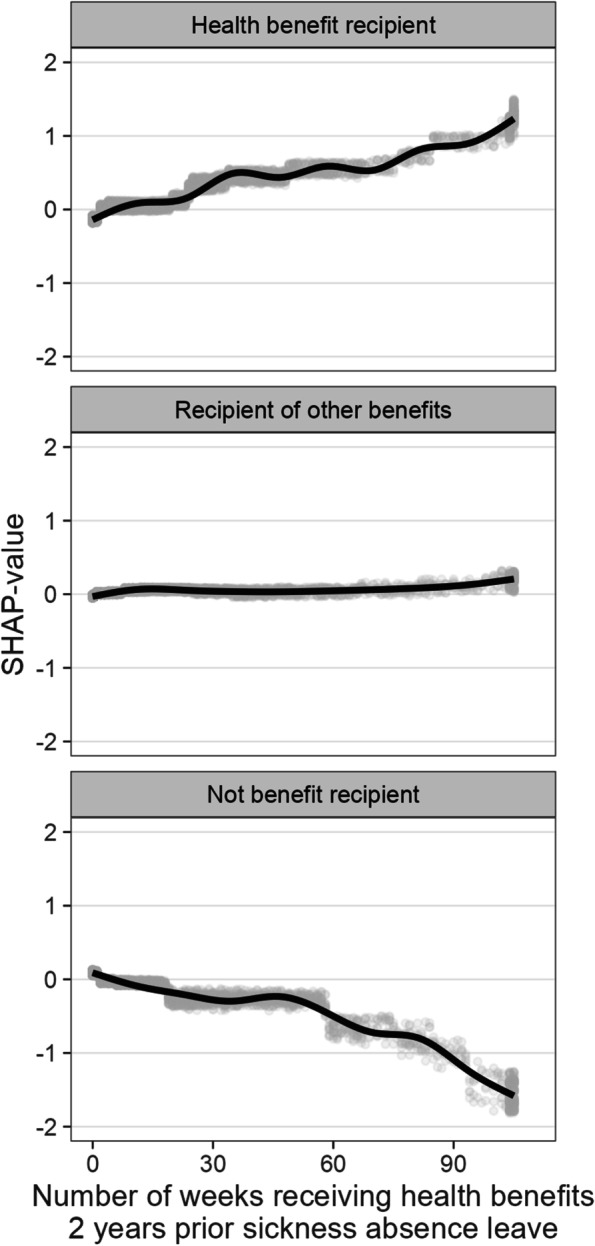
SHAP dependence plots at 1 year follow-up

For the data mining section, we focus on prediction patterns derived from XGB that conflict modelling using MLR. Figure 5 shows the SHAP values in the test set at 1 year follow-up for the variable, indicating the number of weeks with health benefits the two years before sickness absence leave. The dense grey cloud in each figure displays the SHAP value for all observations. The clouds show a narrow distribution of the SHAP values around each week. A cubic spline smooths the relationship between the number of weeks and the assigned SHAP value to predict one of the three outcomes. Additionally, the figures show that an increasing number of weeks increases the associated SHAP value (risks) of being a health benefit recipient at follow-up. Conversely, an increase in the number of weeks decreases the SHAP value of not receiving benefits. Weeks on health benefits neither increase nor decrease the risk of being a non-health benefit recipient. This contrasts the MLR-approach (see Table 6), where the number of weeks as health or other benefit recipient is modelled as a step-wise effect by grouping the variables into three categories.

**Table 6:** Multinomial logistic regression: 1 year follow-up

Outcome (Ref.: Not benefit recipient)	**Health benefit recipient**			**Recipient of other benefits**		
	**OR**	**95% CI**	***P*** **-value**	**OR**	**95% CI**	***P*** **-value**
(Intercept)	0.1	[0.07; 0.14]	< 0.01	0.11	[0.08; 0.14]	< 0.01
Job skill level (Ref.: Basic)						
Highest	0.87	[0.71; 1.06]	0.17	0.92	[0.76; 1.1]	0.35
Medium	0.89	[0.77; 1.04]	0.14	0.9	[0.79; 1.04]	0.15
Few or no	0.94	[0.77; 1.14]	0.51	1.09	[0.91; 1.3]	0.34
Unstated	0.93	[0.75; 1.15]	0.5	1.02	[0.83; 1.24]	0.88
Industry (Ref.: Human health and social work activities)						
Accommodation and food service activities	0.91	[0.64; 1.28]	0.59	0.99	[0.72; 1.36]	0.95
Agriculture	1.19	[0.7; 2.03]	0.53	0.86	[0.5; 1.49]	0.59
Construction	0.9	[0.7; 1.16]	0.43	0.88	[0.69; 1.12]	0.3
Courts and prisons, Police, Fire Departments	0.8	[0.45; 1.4]	0.43	0.97	[0.58; 1.61]	0.9
Education	1.05	[0.82; 1.34]	0.71	0.98	[0.78; 1.24]	0.86
Manufacturing, mining and quarrying	1.08	[0.88; 1.34]	0.46	0.96	[0.78; 1.18]	0.69
Other branches	0.95	[0.8; 1.12]	0.53	1.06	[0.91; 1.23]	0.47
Public administration	0.92	[0.66; 1.29]	0.62	1.19	[0.89; 1.6]	0.25
Transporting and storage	0.97	[0.75; 1.26]	0.83	1.02	[0.8; 1.3]	0.89
Unstated	1.15	[0.52; 2.58]	0.73	1.24	[0.59; 2.63]	0.57
Wholesale and retail trade, repair of motor vehicles and motorcycles	1.06	[0.88; 1.29]	0.52	1.08	[0.9; 1.3]	0.39
Age groups (Ref.: 20–29)						
30–39	1.27	[1.07; 1.51]	0.01	1.09	[0.93; 1.27]	0.27
40–49	1.47	[1.24; 1.75]	< 0.01	1.25	[1.07; 1.46]	0.01
50–54	2.22	[1.8; 2.73]	< 0.01	1.3	[1.06; 1.59]	0.01
Women (Ref.: Men)	1.06	[0.93; 1.21]	0.37	1.14	[1.01; 1.29]	0.03
Not Danish Citizenship (Reference: Danish Citizenship)	1.18	[0.93; 1.5]	0.17	1.48	[1.19; 1.83]	< 0.01
Family type (Reference: Couple with resident children)						
Couple without resident children	1.23	[1.04; 1.47]	0.02	1.08	[0.91; 1.27]	0.39
Single with resident children	1.44	[1.21; 1.72]	< 0.01	1.63	[1.4; 1.92]	< 0.01
Single without resident children	1.34	[1.16; 1.54]	< 0.01	1.48	[1.3; 1.69]	< 0.01
No unemployment insurance (Ref.: Insured)	1.11	[0.94; 1.32]	0.22	1.11	[0.95; 1.3]	0.2
Unemployed (Ref.: Employed)	1.96	[1.67; 2.31]	< 0.01	1.75	[1.5; 2.03]	< 0.01
Reason Sickness Absence (Ref.: Stress/Burnout)						
Depression	2	[1.75; 2.27]	< 0.01	1.64	[1.45; 1.84]	< 0.01
Mental ill health without further specification	2.57	[2.14; 3.09]	< 0.01	1.48	[1.23; 1.78]	< 0.01
Anxiety	2.14	[1.61; 2.83]	< 0.01	1.48	[1.12; 1.95]	0.01
Number of weeks receiving health related benefits 2 years prior sickness absence event (Ref.: 0 weeks)						
1–26	1.43	[1.26; 1.62]	< 0.01	1.34	[1.19; 1.51]	< 0.01
> 26	3.64	[3.01; 4.4]	< 0.01	2.01	[1.65; 2.45]	< 0.01
Number of weeks receiving other benefits 2 years prior sickness absence event (Ref.: 0 weeks)						
1–26	1.33	[1.14; 1.55]	< 0.01	1.86	[1.62; 2.14]	< 0.01
> 26	2.06	[1.73; 2.46]	< 0.01	3.2	[2.73; 3.76]	< 0.01
Time passed between the first day of sickness absence and the baseline visit at the jobs and benefits office (Ref.: 0–30 days)						
31–60	0.85	[0.71; 1.03]	0.09	1.01	[0.84; 1.21]	0.94
> 60	0.93	[0.76; 1.13]	0.45	1.22	[1.01; 1.48]	0.04
Participation in the Danish RTW program						
Intervention	1.05	[0.89; 1.24]	0.55	1.36	[1.16; 1.59]	< 0.01
Not eligible	0.73	[0.62; 0.84]	< 0.01	0.74	[0.64; 0.86]	< 0.01
Period: Before 2012 (Ref.: 2012)	1.02	[0.89; 1.18]	0.74	1.1	[0.97; 1.26]	0.14

*OR* Odds Ratio, *CI* Confidence interval, *N* = 9.752

**Fig. 6:**
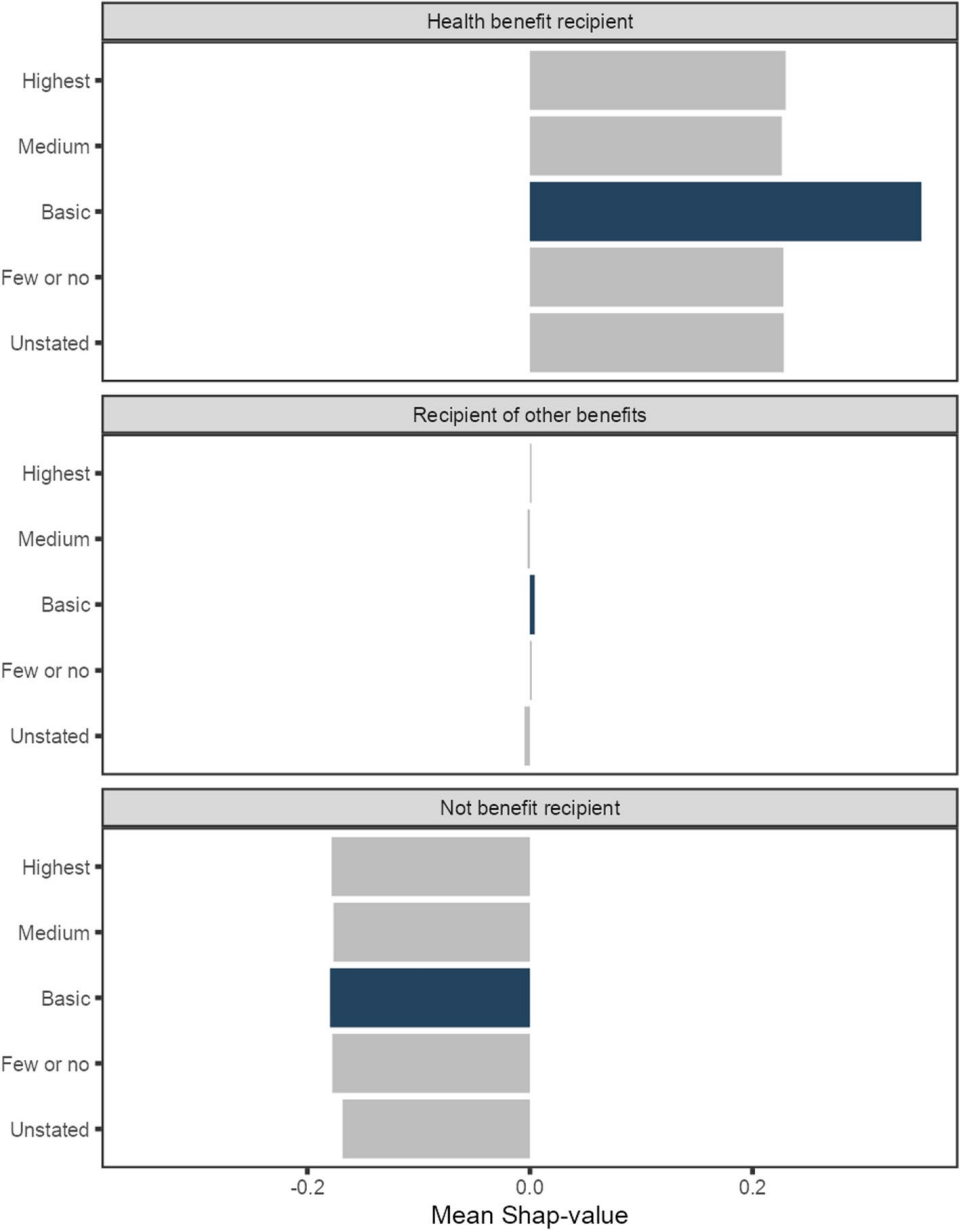
SHAP values by outcome and job skill level for self-reported “mental ill-health without further specification”

Figure 6 shows an interesting pattern for the mean SHAP values for self-reported “mental ill-health without further specification” by job skill level. The SHAP values are markedly higher for persons with a basic job than other job skill levels for the health benefit outcome. Contrary, the SHAP value for basic job skill level is not higher than other job skill categories when we assess the mean SHAP value for other self-reported reasons for sickness absence (figures not shown). Therefore, the analysis of SHAP values suggests an interaction effect between basic job skill level and self-reported “mental ill-health without further specification”. Moreover, this interaction predicts a higher risk of being a health benefit recipient at follow-up.

## Discussion

MLR and XGB showed similar performance in predicting welfare dependency from mental health-related sickness absence at follow-up after 1, 3, and 5 years. The scaled-Brier scores indicate that the prediction models improve the predictive performance by 0.08–0.15 over average probabilities. While this represents an improvement over average probabilities, this improvement is modest. Industry and job skills, along with citizenship, unemployment insurance, gender and period, had limited importance in predicting welfare dependency in both models. Welfare dependency history and reason for sickness absence were, on the other hand, strong predictors. Finally, SHAP analysis demonstrated that XGB and MLR relied on different predictive associations. In particular, XGB used a linear or curvilinear relationship to model the association between welfare dependency and the number of weeks receiving health-related or other types of benefits during the previous two years before sickness absence leave. In contrast, these variables were grouped into three categories in the MLR models. Also, XGB modelled interaction effects, whereas the MLR models only modelled main effects.

We expected XGB's predictive ability to outperform MLR based on the praise for XGB's predictive performance in machine learning competitions across a broad range of prediction problems [24]. In this study, XGB and MLR had a similar predictive ability, although the former model can learn more subtle and non-linear patterns. Thus, the two models' comparative predictive performance suggests that MLR could capture the most important patterns even if XGB were able to model more complex patterns. In other words, with the application of XGB to the same data, we demonstrated that the MLR models capture the most important patterns in the data in predicting welfare dependency. A Danish study also found a similar predictive performance of RTW of a logistic regression model against a more flexible modelling design combining multistate and survival analysis [32]. However, the sample was not restricted to persons sick-listed with CMD. Two studies [6, 7] makes prediction models with machine learning for RTW using Korean data, but only one of them [6] compares predictive performance across different algorithm, including logistic regression. The study assessing different algorithms showed comparable performance across the algorithms, although the random forest algorithm did slightly better. In sum, along with the existing literature, the present study suggests that flexible machine learning models have limited advantages over traditional models in predicting welfare dependency and the closely related concept of RTW. This would also be in line with both empirical and simulation studies showing that machine learning models do not perform better than traditional models, such as logistic regression in clinical prediction modelling, except for settings with a large N [3].

The modest predictive performance of both the MLR and XGB demonstrates that it can be difficult to predict welfare dependency from administrative data. Since the number of observations was reasonably large and we tried a very flexible modelling approach, it is unlikely that more observations and other algorithms would improve predictive ability markedly. However, combining administrative data with variables based on self-report may improve predictive performance. For example, satisfaction level with the employer and maintenance of relationship with the company was among the important predictors of RTW across several algorithms in one study [6]. Thus, these predictors, not available from administrative data, could possibly also improve the predictive ability of welfare dependency in our setting.

Datamining showed that MLR might improve performance by modelling some of the predictive associations derived from the SHAP analysis of XGB. For example, XGB demonstrates that the relationship between welfare dependency and the number of weeks as a health or other benefits recipient can be modelled as a curvilinear function to reduce information loss associated with categorizing a continuous variable. Further, SHAP analysis indicated a predictive interaction between basic job skill level and “mental-ill health without further specification”. Due to the nature of data mining, this effect could result from overfitting. Further research assessing similar predictive interaction effects could establish whether this interaction is consistent across data in new settings. We did not find any strong indications of other interactions in the data mining. This corroborates the finding that XGB cannot achieve better predictive performance than the MLR even if XGB can deal with interaction effects that are not pre-specified in the model.

In practice, prediction models can help job centres in focusing efforts on workers with a high risk of persistent welfare dependency rather than “wasting” scarce resources on individuals that are likely to return to work by themselves. In addition, it may help to set expectations on risk for welfare dependency that are more realistic for both workers and job centres to ease disappointment if coming off welfare dependency proves difficult. Here the use of administrative data can be useful because it can provide consistent and objective information, whereas self-report can suffer from different biases (e.g. difficulty in remembering the exact number of months receiving benefits the past 24 months.). A downside to administrative data is that it will not always be up to date at the time of prediction. In these circumstances, the prediction model must rely on data with some lag, which may reduce the models’ predictive ability. Prediction models can be implemented with software that can extract information from administrative data and by typing in self-reports to the software. The web page https://qrisk.org/three provides an example of an interface where the user types in information to get a risk score of a person’s risk of developing a heart attack or stroke in the next 10 years based on scientific studies [33].

The present study has demonstrated how flexible machine learning modelling can complement traditional statistical methods. However, researchers should also weigh benefits against potential costs and challenges. Below we list some considerations:

1. The additional application of machine learning models to traditional statistical models is more demanding of other researchers with limited knowledge of machine learning models. This is likely a problem in occupational health research, where most researchers have limited machine learning training. However, occupational health researchers are vital in providing insights into the data being used for prediction modelling. We note that applied textbooks in prediction modelling all stress the value of expert knowledge in variable selection [3, 4, 31]. Thus, the application of machine learning models is ideally completed through partnerships between domain experts and data scientists.
2. Interpreting flexible machine learning models using explanatory algorithms like SHAP is a relatively new field of research. This further raises the barriers to other researchers assessing the reported predictive patterns of flexible machine learning models.
3. The application of machine learning models in occupational health research would also benefit from a more structured approach to data mining. While textbooks cover different algorithms, e.g. [34, 35], strategies or guidelines based on a consensus among experts would help researchers use more structured approaches to data mining.

## Conclusions

Flexible machine learning models like XGB can supplement traditional statistical methods like MLR in occupational health research by providing a benchmark for predictive performance and traditional statistical models' ability to capture important associations for a given set of predictors as well as potential violations of linearity. For example, considerable differences in performance could indicate that a traditional statistical model has failed to model important non-linearities or has too extensive information loss from categorizing continuous variables. In this case, SHAP analysis of flexible machine learning models using variable importance and SHAP-dependence plots can help detect important associations.

## Data Availability

The data and code that support the findings of this study are available from Statistics Denmark, but restrictions apply to the availability of these data, which were used under license for the current study, and so are not publicly available. Data and code are however available from the corresponding author (Søren Skotte Bjerregaard, ssk@nfa.dk) upon reasonable request and with permission of Statistics Denmark, account number 708291.

## Abbreviations

RTW: Return to Work
XGB: XGboost
MLR: Multinomial logistic regression
SHAP: SHapley Additive eXplanation
AUC-ROC: Area under the Curve Receiver Operating Characteristics
CMD: Common mental disorders
LOCF: Last Observation Carried Forward
